# Supplementary material for: Predation and fragmentation portrayed in the statistical structure of prey time series
Source: BMC Ecol. 2009 May 6;9:10. doi: 10.1186/1472-6785-9-10 (PMC2689204; doi:10.1186/1472-6785-9-10)
Supplement: Additional file 2 — Voles and related classes ODDox Documentation. ODDox documentation of the agent-based model (ALMaSS) applied by Hendrichsen et al. The documentation is started by activating main.html. [file 1472-6785-9-10-S2.zip › Vole_ODDox/functions_func_0x66.html]

ALMaSS ODDox: Class Members - Functions

- Main Page
- Related Pages
- Classes
- Files

- Alphabetical List
- Class List
- Class Hierarchy
- Class Members

- All
- Functions
- Variables

- a
- b
- c
- d
- e
- f
- g
- h
- i
- k
- l
- m
- n
- o
- p
- r
- s
- t
- u
- v
- w
- ~

### - f -

- FA\_AmmoniumSulphate()
  : Farm- FA\_GreenManure()
    : Farm- FA\_Manure()
      : Farm- FA\_NPK()
        : Farm- FA\_PK()
          : Farm- FA\_Sludge()
            : Farm- FA\_Slurry()
              : Farm- Farm()
                : Farm- FarmEvent()
                  : FarmEvent- FileAppendOutput()
                    : probe\_data- FileOutput()
                      : probe\_data- FindClosest()
                        : Population\_Manager- FindClosestFemale()
                          : Vole\_Population\_Manager- FindClosestMale()
                            : Vole\_Population\_Manager- FindRandomMale()
                              : Vole\_Population\_Manager- FledgelingProbeOutput()
                                : Population\_Manager- FP\_GreenManure()
                                  : Farm- FP\_LiquidNH3()
                                    : Farm- FP\_ManganeseSulphate()
                                      : Farm- FP\_Manure()
                                        : Farm- FP\_NPK()
                                          : Farm- FP\_NPKS()
                                            : Farm- FP\_PK()
                                              : Farm- FP\_Sludge()
                                                : Farm- FP\_Slurry()
                                                  : Farm- FreeLocation()
                                                    : Vole\_Female
                                                    , Vole\_Base
                                                    , Vole\_Male- FungicideTreat()
                                                      : Farm

---

Generated on Thu Jan 22 14:13:45 2009 for ALMaSS ODDox by 
 1.5.6
